# Immunoglobulin G Trough Levels and Infection Risk in Adults with Inborn Errors of Immunity Receiving Immunoglobulin Therapy

**DOI:** 10.3390/medicina61091549

**Published:** 2025-08-29

**Authors:** Özge Öztürk Aktaş, Nagihan Orhan Özer, Ceren Kaplankıran, Begüm Görgülü Akın, Betul Ozdel Ozturk, Makbule Seda Bayrak Durmaz, Fikriye Kalkan, Şadan Soyyiğit

**Affiliations:** 1 Department of Immunology and Allergic Diseases, Ankara Bilkent City Hospital, 06800 Ankara, Turkey; nagihanorhanozer@hotmail.com (N.O.Ö.); cerenkaplankiran@gmail.com (C.K.); drbegumgorgulu@gmail.com (B.G.A.); betulozdel84@gmail.com (B.O.O.); dr.seda_bayrak@hotmail.com (M.S.B.D.); fikriyehandan@hotmail.com (F.K.); 2 Division of Allergy and Immunology, Department of Chest Diseases, School of Medicine, Hacettepe University, 06230 Ankara, Turkey; 3 Division of Allergy and Clinical Immunology, Ankara Bilkent City Hospital, School of Medicine, Ankara Yildirim Beyazit University, 06800 Ankara, Turkey; sadansoyyigit@hotmail.com

**Keywords:** hypogammaglobulinemia, IgG trough levels, adult primary immunodeficiency

## Abstract

*Background and Objectives:* Inborn errors of immunity are increasingly diagnosed in developing countries. Immunoglobulin replacement therapy (IGRT) remains the cornerstone of treatment in these patients, and its monitoring has gained importance for optimizing outcomes. We conducted a retrospective study to evaluate the relationship between IgG trough levels and infections in adults with inborn errors of immunity receiving IGRT. *Materials and Methods:* Adults diagnosed with primary immunodeficiency and receiving IGRT for at least six months were included. Serum IgG trough levels were measured, and infections during follow-up were systematically recorded. *Results:* A total of 31 adult patients (CVID: 29, XLA: 2) were analyzed. The mean follow-up was 13 months, with a mean serum IgG trough level of 815.8 ± 205.9 mg/dL, achieved with an average IGRT dose of 498.8 ± 93.0 mg/kg/month. Dose adjustments were made in 35.5% of patients, mostly due to weight changes and clinical indications. Overall, 74.2% of patients had at least one infection requiring antibiotics. Patients with mean IgG trough levels below 850 mg/dL had significantly higher rates of antibiotic-requiring infections (*p* = 0.032, Mann–Whitney U; mean difference = 0.109, 95% CI: 0.037–0.182; *p* = 0.005 via *t*-test). Multivariate regression confirmed that lower IgG trough levels were independently associated with higher antibiotic-requiring infection rates (B = −0.024, 95% CI: −0.045 to −0.002, *p* = 0.033), while IGRT dose and comorbidities were not significant. *Conclusions:* IGRT plays a key role in reducing antibiotic-requiring infections in patients with primary immunodeficiency. Regular monitoring and individualized dose adjustments may help optimize outcomes. Further prospective studies are needed to confirm these findings.

## 1. Introduction

Inborn errors of immunity (IEIs), previously referred to as primary immunodeficiency diseases (PID), present clinically with increased susceptibility to infections, autoimmune diseases, autoinflammatory disorders, allergies, bone marrow failure, and cancer [1,2]. Management varies by severity and is fundamentally based on the specific immune defect. A precise diagnosis—especially identifying primary antibody deficiency (PAD), which accounts for 50–70% of cases—is essential to guide tailored screening, targeted therapies, immunoglobulin replacement, and curative interventions like bone marrow transplantation or gene therapy where appropriate [3,4]. Immunoglobulin therapy is utilized in a range of immunodeficiencies, including primary antibody deficiencies, severe combined immunodeficiency (SCID), and other combined immunodeficiencies, particularly during the period preceding the restoration of B cell function, as well as in specific conditions characterized by defective antibody production or function. In patients with antibody deficiencies, immunoglobulin replacement therapy remains the cornerstone of management, with sustained and regular administration significantly reducing morbidity and mortality [5,6]. The lack of ongoing therapy may lead to life-threatening outcomes [7]. Intravenous immunoglobulin G (IVIG) replacement is typically administered at doses of 300–800 mg/kg every 3–4 weeks, with infusion rates ranging from 0.01 to 0.08 mL/kg/min, depending on patient tolerance. The effectiveness of therapy is evaluated based on steady-state IgG trough levels in conjunction with the patient’s clinical response, as outlined in current practice guidelines [8]. The optimal IgG trough level remains unclear. While earlier studies suggested that levels above 500 mg/dL may offer protection against severe infections, more recent recommendations propose higher targets, ranging from 600 to 1000 mg/dL [9,10]. Although some meta-analyses have reported that higher IgG trough levels are associated with reduced infection rates, other studies found this correlation only with subcutaneous immunoglobulin (SCIG) trough levels—not with IVIG trough levels [11,12]. Conflicting results in the literature regarding optimal IgG trough levels may be attributed to several factors. These include heterogeneity among patients with primary immunodeficiency, such as differences in underlying diagnoses, severity of antibody deficiency, and presence of comorbidities [13,14]. Variations in the route of administration (e.g., IVIG vs. SCIG) and dosing schedules may also affect pharmacokinetics and achieved serum levels [11,12]. Additionally, differences in outcome definitions (e.g., total infections vs. pneumonia) and retrospective study designs may contribute to inconsistencies [15]. Collectively, these factors likely explain the lack of consensus on a universally accepted IgG target level and emphasize the importance of individualized therapeutic strategies. Despite the widespread use of IGRT, the optimal monitoring parameters and infection-related outcome measures remain a subject of ongoing debate. In particular, there is a lack of real-world data evaluating the relationship between IgG trough levels and infection risk in adult patients with inborn errors of immunity.

This study aimed to investigate the association between IgG trough levels and infection outcomes in adult patients with primary immunodeficiency receiving immunoglobulin replacement therapy, in order to better understand the potential clinical impact of varying trough levels within our cohort.

## 2. Materials and Methods

### 2.1. Inclusion and Exclusion Criteria

This retrospective study included patients diagnosed with inborn errors of immunity (IEIs) who were receiving regular immunoglobulin replacement therapy (IGRT) at our center and had been followed for at least 6 months between April 2021 and January 2025. A total of 31 patients with hypogammaglobulinemia were enrolled, and their diagnoses were confirmed according to the European Society for Immunodeficiencies (ESID) diagnostic criteria. IGRT was administered once every 3 to 4 weeks. Before each visit, patients were evaluated for infection symptoms, and medications were recorded. Demographic data, family history, clinical findings, and immunological parameters were documented at enrollment. Infections were classified as either major or minor episodes. Those requiring intravenous antibiotic therapy and/or hospitalization, such as pneumonia, sepsis, meningitis, osteomyelitis, septic arthritis, or deep organ abscesses, were defined as major infections. All other infections were considered minor and were managed with oral antimicrobials or symptomatic treatment. Lower respiratory tract infection (LRTI) was defined as patients with lower respiratory symptoms but no radiological evidence of pneumonia. Blood samples were collected on the same day or one day before the immunoglobulin infusion. Serum IgG, IgA, and IgM levels were measured by using the Siemens BNII nephelometry System (Erlangen, Germany). If an IgG level was obtained during an active infection, it was excluded from the analysis. Only infection-free IgG measurements were included, and the mean IgG trough level was calculated for each patient. Due to variations in follow-up durations among patients, infection rates (infections per patient-months) rather than absolute infection counts were used for analysis to ensure comparability. To achieve a more accurate assessment, a regression analysis was performed to evaluate the association between mean IgG trough levels and infection rates, adjusted for comorbidities.

### 2.2. Statistical Analysis

Statistical analyses were performed using IBM SPSS Statistics version 27 (IBM Corp., Armonk, NY, USA). The normality of continuous variables was assessed using the Shapiro–Wilk test. Parametric data are presented as mean ± standard deviation (SD), while non-parametric data are presented as median (interquartile range, IQR) or range, as appropriate. Categorical variables are summarized as frequencies and percentages. For comparisons between two groups, the independent samples *t*-test or Mann–Whitney U test was used, depending on data distribution. The chi-square or Fisher’s exact test was used for categorical comparisons. Associations between continuous variables were evaluated using Spearman’s rank correlation due to non-normal distribution [16].

A multivariate linear regression analysis was performed to assess the independent effect of mean IgG trough levels on the antibiotic-requiring infection rate (per patient-month), adjusting for immunoglobulin dose (mg/kg/month) and the presence of comorbidities. The results are reported with unstandardized regression coefficients (Bs), corresponding 95% confidence intervals (CIs), and *p*-values. A two-tailed *p*-value of <0.05 was considered statistically significant. The study was approved by the Ankara City Hospital Ethics Committee (Approval No: TABED 1-25-979; Date: 12 February 2025). All data were retrospectively analyzed in anonymized form, and no identifiable personal information was collected or reported.

## 3. Results

### 3.1. Patient Characteristics

The study population consisted of 31 patients, including 12 females (38.7%) and 19 males (61.3%), with a mean age of 36.4 years, SD ± 14.2. Among the study population, two patients were diagnosed with X-linked agammaglobulinemia and twenty-nine patients with common variable immunodeficiency (CVID). The median follow-up duration was 13 months, with a minimum of 6 months and a maximum of 72 months. The mean age at diagnosis was 28.1 years (SD: 17). Among the 31 patients included in the study, 74.2% had at least one comorbid condition. The most common comorbidities were autoimmune diseases (58.1%) and bronchiectasis (41.9%), followed by asthma (16.3%), inflammatory bowel disease (16.1%), and lymphoma (6.5%). Notably, 25.8% of patients had no identifiable comorbidities. (Table 1)

### 3.2. Immunoglobulin Therapy and Dose Adjustments

At the time of initial assessment, the patients demonstrated a mean baseline serum IgG level of 366 ± 248 mg/dL, highlighting the degree of hypogammaglobulinemia prior to the initiation of immunoglobulin replacement therapy. Changes in IGRT dosage were recorded in 11 out of 31 patients (35.5%). The most common reasons for dosage adjustment were weight gain (five patients, 16.1%), low IgG trough levels (two patients, 6.5%), frequent infections (two patients, 6.5%), and the diagnosis of lymphoma combined with weight gain (one patient, 3.2%). In one patient (3.2%), the dose was increased specifically due to a low IgG trough level measured at 7.3 g/L. Malignancy was identified as the reason for dose adjustment in another patient (3.2%). No dosage changes were made in 20 patients (64.5%).

### 3.3. Infection Outcomes and Association with IgG Levels

**Figure 1:**
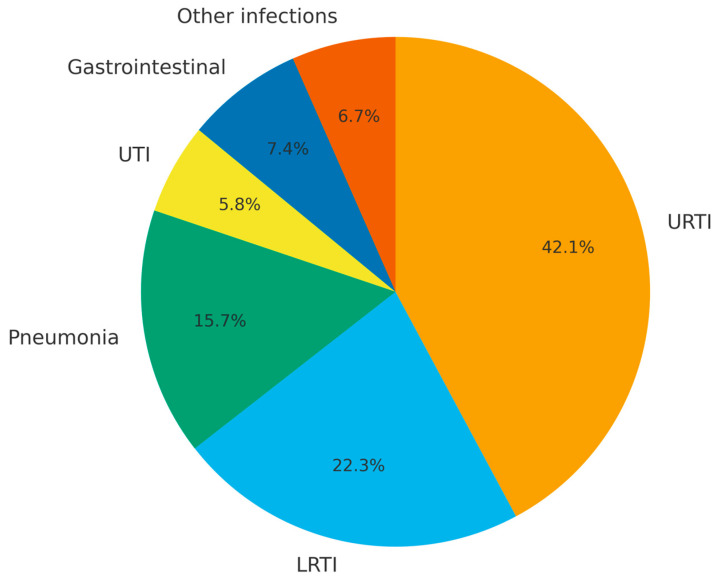
Distribution of infections by type during the total follow-up period among patients receiving immunoglobulin replacement therapy. URTI: upper respiratory tract infection; LRTI: lower respiratory tract infection; GIS: gastrointestinal system; UTI: urinary tract infection.

The mean serum IgG trough level was 815.8 ± 205.9 mg/dL, which was achieved with a mean IGRT dose of 498.8 ± 93.0 mg/kg/month. A total of 121 infections were recorded during the total follow-up period. URTI occurred in 51 cases (42.1%), LRTI in 27 cases (22.3%), pneumonia in 19 cases (15.7%), UTI in 7 cases (5.8%), gastrointestinal infections in 9 cases (7.4%), and other infections in 8 cases (6.7%). Infections presented in the chart were identified in a cohort of 31 patients over a median follow-up duration of approximately 13 months (Figure 1).

Out of 31 patients, 14 (45.2%) experienced at least one major infection during the follow-up period, while 17 patients (54.8%) had no history of major infections.

There was no statistically significant difference in IgG trough levels between patients with and without major infections.

During the follow-up period, 8 out of 31 patients (25.8%) did not experience any antibiotic-requiring infections. Among those who did, 19.4% had one episode, 29.0% had two episodes, 16.1% had three episodes, and 9.7% experienced four episodes. These findings indicate that approximately three-quarters (74.2%) of patients required at least one course of antibiotics due to infection.

**Figure 2:**
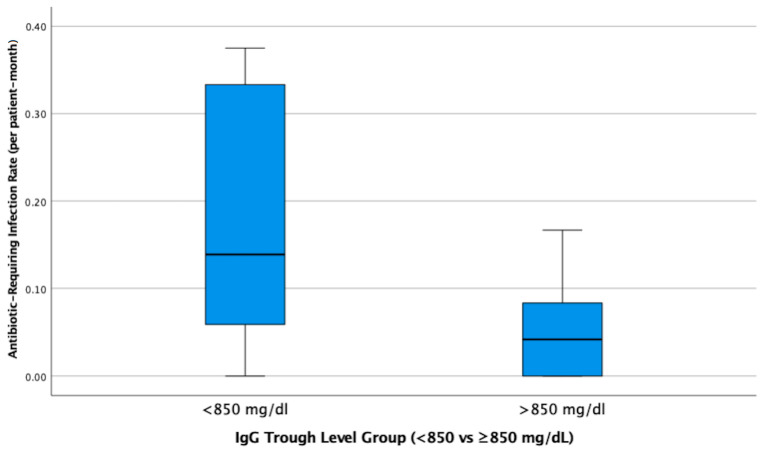
Boxplot comparing the antibiotic-requiring infection rate (per patient-month) between patients with mean IgG trough levels below and above 850 mg/dL. A statistically significant difference was observed (*p* = 0.032, Mann–Whitney U; mean difference = 0.109, 95% CI: 0.037–0.182; *p* = 0.005 via *t*-test).

Patients with mean serum IgG trough levels below 850 mg/dL had significantly higher rates of antibiotic-requiring infections compared to those with higher levels (*p* = 0.032, Mann–Whitney U test). For effect size interpretation, a supplementary independent samples *t*-test was performed, showing a mean difference of 0.109 (95% CI: 0.037–0.182; *p* = 0.005) (Figure 2).

**Figure 3:**
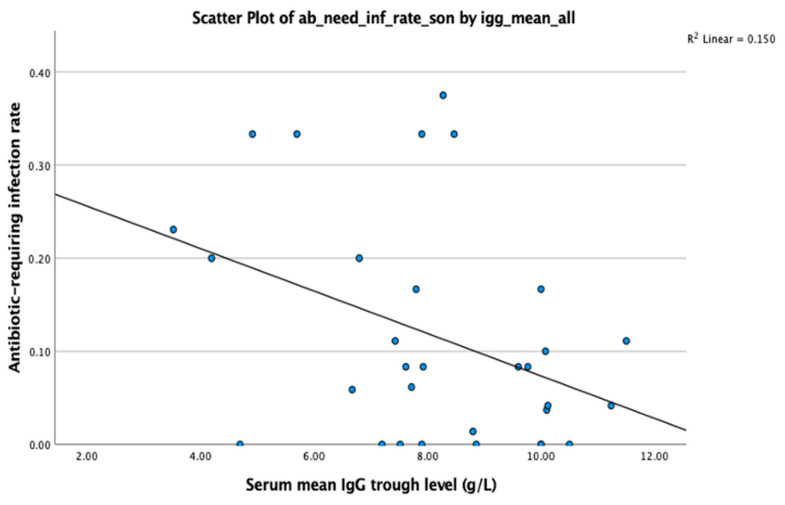
Association between serum IgG trough level and antibiotic-requiring infection rate. Linear regression revealed a significant inverse correlation between IgG trough level and antibiotic-requiring infection rate (*p* = 0.033).

A multivariate linear regression analysis was performed to evaluate the association between mean serum IgG trough level and antibiotic-requiring infection rate (per patient-month), adjusting for immunoglobulin dose (mg/kg/month) and the presence of comorbidities. The model revealed that only IgG trough level was significantly associated with the antibiotic-requiring infection rate (B = −0.024, 95% CI: −0.045 to −0.002, *p* = 0.033), while immunoglobulin dose (*p* = 0.527) and comorbidity status (*p* = 0.943) did not show significant effects. Notably, Spearman’s correlation between IgG and infection rate did not reach statistical significance (ρ = −0.297, *p* = 0.105), suggesting that the observed effect of IgG in the regression model may reflect its independent contribution when adjusted for confounders (Figure 3).

## 4. Discussion

IgG trough levels are commonly used to evaluate the adequacy of immunoglobulin replacement therapy in patients with primary immunodeficiency. While higher trough levels are generally associated with reduced infection risk, the optimal target remains uncertain [8]. Nonetheless, the optimal IgG trough level for achieving maximal benefit remains ambiguous. Several studies have recommended a target IgG trough level above 500 mg/dL for optimal infection prevention, with some suggesting a range between 600 and 900 mg/dL [9,17]. Additionally, other reports have indicated that maintaining levels between 650 and 1000 mg/dL may also be effective in reducing infection risk [10]. In our study, the mean serum IgG trough level was 815.8 ± 205.9 mg/dL, achieved with a mean immunoglobulin dose of 498.8 ± 93.0 mg/kg/month. These results are similar to a systematic review of 28 studies involving 1218 patients, which reported that IVIG doses ranging from 387 to 560 mg/kg every 3 to 4 weeks yielded mean IgG trough levels between 660 and 1280 mg/dL [13]. In another study investigating the patients with X-linked agammaglobulinemia (XLA), a cohort of 11 patients receiving a mean IVIG dose of 414 mg/kg/month achieved a mean trough IgG level of 435 mg/dL [18]. This relatively low level is consistent with the complete absence of endogenous immunoglobulin production in XLA, where patients are entirely dependent on exogenous replacement. The lower levels observed in XLA reflect the absence of endogenous IgG production, in contrast to CVID patients who may retain partial immunoglobulin synthesis, explaining the relatively higher trough levels in our cohort.

Several studies have reported several associations between (IVIG) dose and serum trough IgG levels in patients with PID. In a retrospective analysis by Khan et al., no significant correlation was found between the annual IVIG dose and serum trough IgG levels, suggesting that body-size-based dosing may not reliably predict achieved IgG concentrations [19]. Moreover, Suri et al. observed that increasing the IVIG dose by 100 mg/kg resulted in a modest mean increase in serum IgG (~53.6 mg/dL), yet this change did not translate into a reduction in infection rates, highlighting the complexity of optimizing replacement therapy based on serum levels alone [18]. A previous study demonstrated that the dose of subcutaneous immunoglobulin had a significant impact on serum IgG trough levels, whereas patient-specific factors such as body mass index (BMI) had limited predictive value [20]. In our study, no significant link was observed between the provided immunoglobulin dosage and the resultant IgG trough levels. The lack of correlation in our data may reflect individual differences in IGRT bioavailability, comorbidities, immunoglobulin loss, or catabolism—factors that require further investigation. The limited sample size of our cohort may explain this finding.

In patients with primary immunodeficiency receiving intravenous immunoglobulin therapy, increasing IgG trough levels up to 960 mg/dL has been associated with improved clinical outcomes and a reduced risk of infections [13]. Furthermore, a meta-analysis demonstrated that pneumonia incidence declined by 27% for every 100 mg/dL increase in trough IgG levels. These findings suggest a progressive reduction in pneumonia risk with rising IgG trough levels, at least up to 1000 mg/dL [11]. However, various studies in the literature have reported inconsistent associations between IgG trough levels and infection frequency. Shrestha et al. conducted a systematic review and meta-analysis including multiple studies involving patients with primary immunodeficiency (PID) and found no significant association between higher IVIG trough levels and a reduced rate of infections [12]. In this study, we evaluated the relationships between IgG trough levels and multiple clinical infection outcomes, including overall infection rates, the incidence of infections requiring antibiotics, pneumonia incidence, total infection incidence, and major infection incidence. Among these, a statistically significant association was found only between IgG trough levels and the incidence of antibiotic-requiring infections. The fact that only antibiotic-requiring infections showed a significant inverse correlation with IgG trough levels, while other subgroups did not, may be due to the bacterial nature of these infections. Minor infections are often viral and self-limiting, and thus less affected by IgG replacement therapy, as also noted by Lucas et al. and Orange et al. [11,15]. This discrepancy may reflect the heterogeneity of infection severity and the retrospective nature of data collection, which may have led to under-reporting or the misclassification of minor or major infections. Furthermore, the relatively small sample size might have limited the statistical power to detect differences in these secondary outcomes. These findings suggest that while IgG trough levels are useful in predicting clinically significant infection risk requiring treatment, they may not fully capture the broader spectrum of infectious burden. Moreover, antibiotic use is likely a more objective marker of clinically relevant bacterial infections, while mild episodes may be under-reported due to recall bias. The multivariate regression confirmed this significant inverse association after adjusting for immunoglobulin dose and comorbidities, suggesting that factors such as body weight, protein loss, or other comorbid conditions may substantially influence the relationship between IgG levels and infection outcomes.

Lucas et al. demonstrated that, over an initial two-decade period, increasing the mean IgG trough level from 644  to 828 mg/dL was associated with a decrease in infection rates from 2.8  ±  3.0 to 1.9  ±  1.9 infections per patient-year. However, they also noted that, in the following decade, infection rates stabilized at 2.3  ±  2.0 infections per patient-year despite achieving even higher mean trough levels (1006  ±  246 mg/dL) [15]. Collectively, these findings support individualized IgG dosing strategies aiming for a trough level of at least 700–800 mg/dL to achieve optimal protection against infections in patients with predominant antibody deficiencies [13]. In our study, patients with IgG trough levels above 850 mg/dL had lower rates of antibiotic-requiring infections, consistent with some prior reports. However, given that other studies have failed to show a clear association between infection frequency and IgG trough levels and that our own analysis did not demonstrate a consistent relationship across all infection subgroups these findings should be interpreted with caution.

To contextualize our findings, the previous literature has reported both supportive and conflicting evidence regarding the relationship between IgG trough levels and infection risk in patients with primary immunodeficiencies. A systematic review published in 2021 highlighted an inverse association between IgG trough levels and infection risk, suggesting a potential dose–response relationship; however, the meta-regression data, particularly in pediatric populations, remain limited [13]. One observational study evaluating the timing of infections in patients receiving intravenous immunoglobulin found that infections tended to cluster toward the end of the infusion cycle, supporting the importance of maintaining stable serum IgG levels throughout the dosing interval [21]. Additional reports have emphasized the need for individualized immunoglobulin dosing strategies, particularly in relation to clinically meaningful outcomes such as pneumonia prevention [22]. In subcutaneous immunoglobulin therapy settings, analyses have demonstrated that maintaining higher steady-state IgG levels is associated with lower infection rates [23].

Our finding—that IgG trough levels are inversely associated with the incidence of antibiotic-requiring infections but not with overall or major infection rates—appears consistent with these heterogeneous observations. This suggests that the clinical impact of trough levels may be influenced by variables such as the route of administration (IVIG vs. SCIG), temporal vulnerability to infection, and individual patient characteristics. Taken together, the available evidence underscores the relevance of individualized IgG trough targets based on patient-specific risk profiles and clinically significant endpoints. Furthermore, comorbidities such as bronchiectasis, autoimmune diseases, and hematological complications may further increase infection risk in patients with primary immunodeficiencies by impairing mucosal barriers or immune regulation. Studies have shown that both structural lung damage and immune dysregulation contribute to heightened susceptibility to infections, suggesting that comorbidity burden should be considered when interpreting the association between IgG trough levels and clinical outcomes [14,24]. Regular monitoring of trough levels combined with individualized dose adjustments may help optimize outcomes, especially in patients with a high infection burden or significant comorbidities. Importantly, this study was not designed to establish a specific optimal IgG trough level. Rather, our aim was to evaluate the association between IgG levels and infection outcomes within our real-life cohort to contribute to the understanding of individualized treatment needs.

The strength of this study lies in its evaluation of a very important and unique population, providing valuable information for immunodeficiency patients receiving immunoglobulin replacement therapy and being monitored for IgG trough levels. Also, this study contributes real-world data from an adult IEI cohort—an under-represented population in previous research—focusing on the association between IgG trough levels and antibiotic-requiring infections. This adult-focused perspective adds novelty to the existing literature and may support individualized follow-up strategies based on clinically relevant outcomes. However, there are several limitations to this study. This study included a relatively small cohort of 31 patients, consisting predominantly of individuals with CVID and a small number with XLA. While both conditions are treated with immunoglobulin replacement therapy, their underlying pathophysiology and clinical expression differ, which may limit the generalizability of our findings. Due to the limited number of XLA cases, subgroup analysis was not feasible. Therefore, the cohort was analyzed collectively based on shared therapeutic principles. Future studies with larger and more homogeneous diagnostic groups will be essential to clarify potential disease-specific associations. Second, the definition of antibiotic-requiring infections is dependent on the physician’s judgment, which may introduce variability. Due to the differences in follow-up duration among patients, infection rates rather than absolute infection counts were calculated and analyzed in order to obtain more objective results. Although some patients experienced dose changes during follow-up, our analysis focused on the mean serum IgG trough level, which reflects the cumulative immunoglobulin exposure over time and is considered the most relevant parameter when evaluating infection risk. Although the number of trough level measurements varied among patients, we used only infection-free values and calculated the mean to reflect steady-state exposure. This method, while subject to some variability, is aligned with published recommendations suggesting that multiple time-point measurements provide a more accurate estimate than a single value [15,22]. Additionally, the retrospective design of the study imposes further limitations. The reliance on historical medical records may have resulted in missing or inconsistently documented data, particularly regarding minor infections or antibiotic prescriptions. Although IgG trough levels measured during active infections were excluded from the analysis, it was not always possible to verify infection status with complete certainty for every time point due to incomplete retrospective documentation. Moreover, outcomes such as antibiotic use and infection categorization were partially dependent on physician documentation, which could introduce bias. No formal consensus-based or standardized criteria were used to adjudicate whether infections warranted antibiotic treatment, and no multi-clinician review was conducted. Thus, inter-observer variability in clinical decision-making may have influenced infection classification. ROC curve analysis to define an optimal IgG trough threshold was not performed due to the small sample size and the limited number of events, which could result in statistically unstable estimates. Future studies with larger cohorts are needed to explore this further. These inherent challenges of retrospective data collection should be taken into consideration when interpreting the study findings.

## 5. Conclusions

This study suggests that immunoglobulin replacement therapy (IGRT) may play an important role in reducing antibiotic-requiring infections in patients with primary immunodeficiency. Rather than aiming to define an optimal IgG trough level, our study focused on evaluating the association between IgG levels and infection outcomes in a real-world clinical setting. However, treatment should always be individualized, as the bioavailability and clinical benefit of IGRT can vary significantly among patients and may not consistently correlate with serum IgG levels. Our recommendation for individualized dose adjustments is not based on a fixed dose–trough expectation but rather on the need for personalized monitoring in patients with ongoing infections or suboptimal IgG levels. Further large-scale studies are necessary to validate these findings before they can be broadly implemented in clinical practice for individualized patient management.

## Figures and Tables

**Table 1:** Baseline demographic and clinical characteristics of the patients.

Parameter	Result
Sex, n (%)	
Female	12 (38.7%)
Male	19 (61.3%)
Mean age, years (SD)	36.4 ± 14.2 Median: 37.5 (range: 19–69)
Diagnosis	
Common variable immunodeficiency (CVID)	29 (93.5%)
X-linked agammaglobulinemia (XLA)	2 (6.5%)
Mean age at diagnosis, years (SD)	28.1 ± 17 Median: 30 (range: 2–63)
Follow-up duration, months	Median: 13 (range: 6–72)
Comorbidities At least one comorbidity	23 (74.2%)
Autoimmune disease	18 (58.1%)
Bronchiectasis	13 (41.9%)
Asthma	5 (16.3%)
Lymphoma	2 (6.5%)
Inflammatory bowel disease	5 (16.1%)
No comorbidity	8 (25.8%)
Baseline IgG level, mg/dL (SD)	366 ± 248
Mean IgG trough level, mg/dL (SD)	815.8 ± 205.9
Mean IVIG dose, mg/kg/month (SD)	498.8 ± 93.0 Median: 477.2 (range: 365.9–692.3)
Infection frequency, n (%)	
At least one major infection	14 (45.2%)
Only minor infections	17 (54.8%)

SD: Standard deviation.

## Data Availability

The authors declare that they have followed the protocols of their work center for the publication of patient data in this study. All data generated or analyzed during this study are included in this article. The data that support the findings of this study are available on request from the corresponding author. The data are not publicly available due to privacy or ethical restrictions.

## Abbreviations

The following abbreviations are used in this manuscript:

IEI	Inborn Errors Of Immunity
PID	Primary Immunodeficiency Diseases
PAD	Primary Antibody Deficiency
SCID	Severe Combined Immunodeficiency
IVIG	Intravenous Immunoglobulin G
SCIG	Subcutaneous Immunoglobulin
ESID	European Society For Immunodeficiencies
CVID	Common Variable Immunodeficiency
XLA	X-linked agammaglobulinemia
